# A novel likely pathogenetic variant p.(Cys235Arg) of the *MEN1* gene in multiple endocrine neoplasia type 1 with multifocal glucagonomas

**DOI:** 10.1007/s40618-023-02287-x

**Published:** 2024-01-31

**Authors:** C. Smirne, G. M. Giacomini, A. M. Berton, B. Pasini, F. Mercalli, F. Prodam, M. Caputo, L. A. A. Brosens, E. L. M. Mollero, R. Pitino, M. Pirisi, G. Aimaretti, E. Ghigo

**Affiliations:** 1 Department of Translational Medicine, University of Piemonte Orientale, Via Solaroli 17, 28100 Novara, Italy; 2 Division of Internal Medicine, University Hospital Maggiore della Carità, 28100 Novara, Italy; 3 Division of Endocrinology, Diabetes and Metabolism, City of Health and Science University Hospital, 10126 Turin, Italy; 4 Department of Medical Sciences, University of Turin, 10126 Turin, Italy; 5 Division of Medical Genetics, City of Health and Science University Hospital, 10126 Turin, Italy; 6 Division of Pathology, University Hospital Maggiore della Carità, 28100 Novara, Italy; 7 Department of Health Sciences, University of Piemonte Orientale, 28100 Novara, Italy; 8 Division of Endocrinology, University Hospital Maggiore della Carità, 28100 Novara, Italy; 9 Department of Pathology, University Medical Center Utrecht, 3584 CX Utrecht, The Netherlands

**Keywords:** Next-generation sequencing, Familiar, Primary hyperparathyroidism, Neuroendocrine pancreatic tumor, Adrenal gland adenoma, Necrolytic migratory erythema

## Abstract

**Purpose:**

Multiple endocrine neoplasia type 1 (MEN1) is a hereditary endocrine syndrome caused by pathogenic variants in *MEN1* tumor suppressor gene. Diagnosis is commonly based on clinical criteria and confirmed by genetic testing. The objective of the present study was to report on a MEN1 case characterized by multiple pancreatic glucagonomas, with particular concern on the possible predisposing genetic defects.

**Methods:**

While conducting an extensive review of the most recent scientific evidence on the unusual glucagonoma familial forms, we analyzed the *MEN1* gene in a 35-year-old female with MEN1, as well as her son and daughter, using Sanger and next-generation sequencing (NGS) approaches. We additionally explored the functional and structural consequences of the identified variant using in silico analyses.

**Results:**

NGS did not show any known pathogenic variant in the tested regions. However, a new non-conservative variant in exon 4 of *MEN1* gene was found in heterozygosity in the patient and in her daughter, resulting in an amino acid substitution from hydrophobic cysteine to hydrophilic arginine at c.703T > C, p.(Cys235Arg). This variant is absent from populations databases and was never reported in full papers: its characteristics, together with the high specificity of the patient’s clinical phenotype, pointed toward a possible causative role.

**Conclusion:**

Our findings confirm the need for careful genetic analysis of patients with MEN1 and establish a likely pathogenic role for the new p.(Cys235Arg) variant, at least in the rare subset of MEN1 associated with glucagonomas.

**Supplementary Information:**

The online version contains supplementary material available at 10.1007/s40618-023-02287-x.

## Introduction

Multiple endocrine neoplasia type 1 (MEN1) is a rare inherited condition (estimated prevalence of 1–10/100,000) characterized by a predisposition to the development of endocrine tumors, mainly involving the parathyroid gland, pancreatic islets and pituitary gland [1, 2]. Most of these tumors are usually benign, but the malignancy of some of them—in particular, carcinoid, islet cell and gastrointestinal tract tumors—is an important cause of mortality [3]. In any case, they can create striking clinical effects because of the secretion of endocrine peptides, such as gastrin, insulin, parathyroid hormone, prolactin, growth hormone, glucagon, or adrenocorticotropic hormone [4]. In this respect, multiple parathyroid tumors causing primary hyperparathyroidism are the most common manifestation of MEN1, with more than 90% penetrance by age 40–50 [4–7].

The disorder, which is transmitted with an autosomal dominant inheritance, is caused by pathogenic variants in the tumor suppressor gene *MEN1*, which encodes a protein named menin. This generally results in an inactivation of the protein function itself [8–11].

To improve life expectancy in patients with MEN1, it would be appropriate to find tumors in their pre-symptomatic phase and promptly refer the patients and their families to a close follow-up with the aim of detecting and treating any other neoplasm at early stage [12]. A pivotal role in this multistep algorithm is currently played by *MEN1* germline genetic testing. As regarding the target population, current guidelines recommend that mutation testing should be offered to index patients with MEN1 and their first-degree relatives, including both those who have clinical manifestations of MEN1 and those who are asymptomatic [1].

As a general rule, all of the above persons who have been offered *MEN1* mutation testing should receive genetic counseling first. Moreover, mutation testing should be performed by a certified clinical genetics laboratory and include the search for point mutations and exon deletion/duplication (Copy Number Variants, CNVs). Finally, all individuals in whom a *MEN1* germline mutation has been detected should be screened indefinitely through adult life for the development of MEN1-associated cancers [13].

In this study, we identified a 35-year-old female with MEN1 bearing in heterozygosity a new variant of *MEN1* gene [c.703T > C, p.(Cys235Arg)]. The same substitution was found also in her young daughter. We then conducted structural in silico analyses of the identified variant, and discussed how it plays a likely pathogenetic biological effect in this disease. Together our results offer a novel piece of information regarding MEN1 genetic testing, with possible practical application in the diagnostic-therapeutic pathway proposed above.

## Materials and methods

### Clinical evaluation of the index patient and genetic analyses

Our research study began with newly diagnosed MEN1 in a patient with relapsed primary hyperparathyroidism. In addition to the diagnostic workup conducted according to current practice guidelines, a mutation analysis was conducted upon obtaining written informed consent.

For this purpose, a blood sample was sent to the genetics laboratory searching for causative germline pathogenic variants related to MEN1. Constitutional DNA was obtained from peripheral blood leukocytes using paramagnetic bead-based capture and purification technology (Maxwell^®^, Promega, Madison, WI, USA). Search for point mutations and copy number variants was performed through next-generation sequencing (NGS) applied to the analysis of the coding regions ± 20 intronic bases flanking the exons of a selected panel of 24 endocrine tumor genes. The latter ones were mainly related to MEN1 (OMIM #131100) and multiple endocrine neoplasia type 4 (MEN4), a rare variant of MEN presenting a MEN1-like phenotype (OMIM #610755) [14]. The library was obtained with SureSelect-Custom Hereditary Cancer Solution (#3294891 probes (Agilent, Santa Clara, CA, USA), testing a total 77 cancer predisposing genes (Online Resource 1).

Confirmation of the results on a second DNA extraction was performed by Sanger sequencing analysis of the fragment in which the variant was identified; then an amplification of this fragment was performed by polymerase chain reaction (PCR) and direct sequencing of the genomic region containing the variant by capillary electrophoresis on the automatic sequencer SeqStudio Genetic Analyzer (Applied Biosystems, Waltham, MA, USA).

The patient had an 11-year-old daughter and a 5-year-old son. Written informed consent was obtained from the parents for mutation analysis in both of them. DNA extracted from their peripheral blood leukocytes was analyzed for the presence of the germline variant previously identified in the mother by Sanger sequency as reported above. Due to the certified tracking of the blood vials, confirmation on a second blood sample was not necessary.

### Structural investigation of the identified variant on the MEN1 protein

Variant interpretation was based on the American College of Medical Genetics and Genomics (ACMG) rules [15] with the support of the Alamut visual software (Sophia Genetics), and VarSome, Franklin and Phyre2/Missense3D informatics tools [16, 17]. The following lines of computational evidence were used to test a possible deleterious effect of the identified variant on the *MEN1* gene product: A-GVGD, BayesDel_addAF, CADD, DANN, DEOGEN2, EIGEN, FATHMM-MKL, LIST-S2, LRT, M-CAP, MetaLR, MetaRNN, MutationAssessor, MutPred, MutationTaster, MVP, PolyPhen2, PrimateAI, PROVEAN, REVEL, SIFT, VARITY.

List of relevant databases and in silico analyses used for the current research study is reported in Online Resource 2 together with their result.

## Results

### Index patient’s clinical findings

**Fig. 1:**
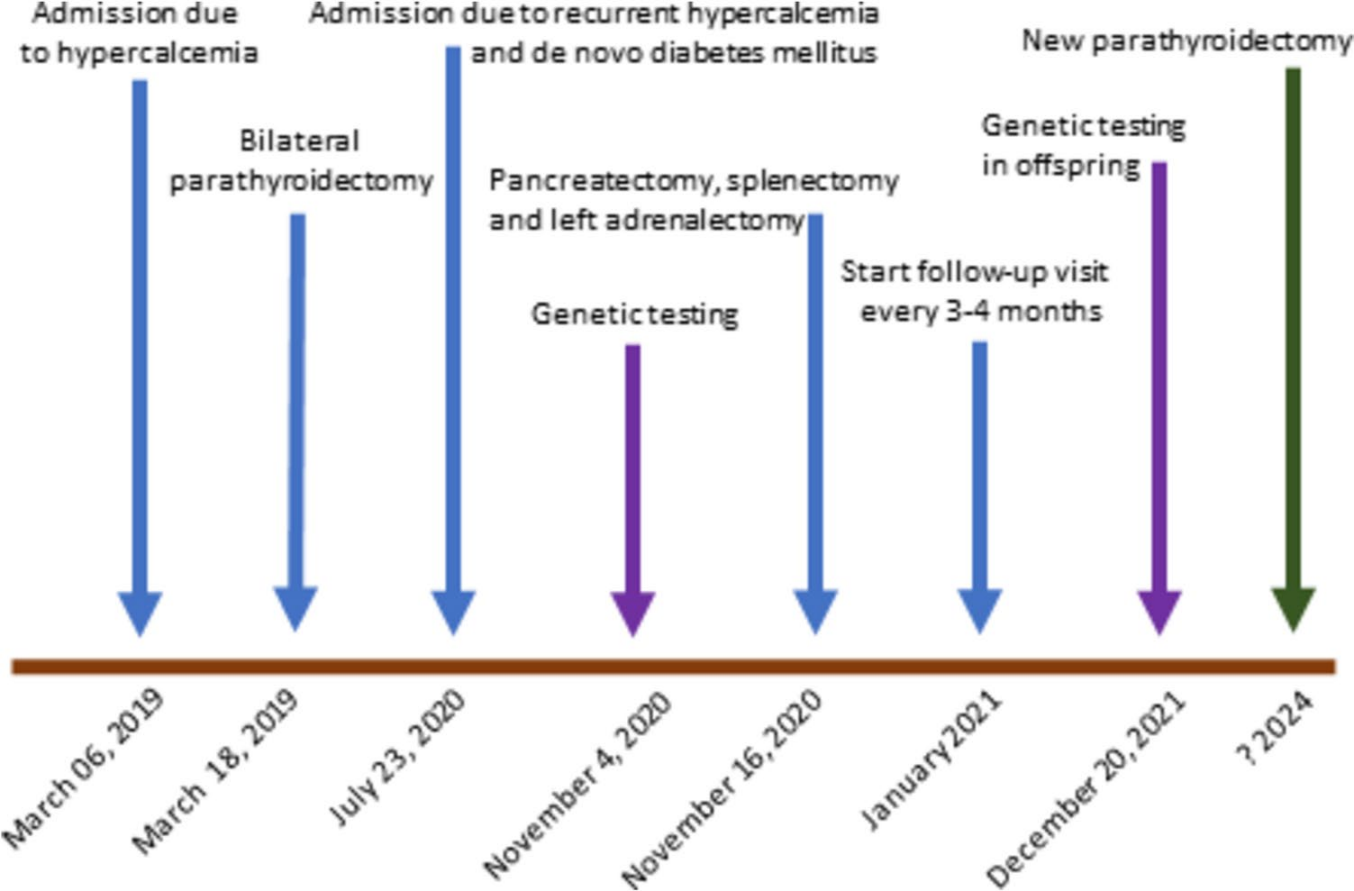
Timeline with relevant data from the clinical history

A 35-year-old woman was admitted to our Department for further evaluation of moderate paucisymptomatic hypercalcemia [total calcium 2.59 mmol/L, local laboratory normal range (NR) 2.15–2.50 mmol/L] and new-onset hyperglycemia [14.3 (NR 3.9–5.6) mmol/L]. The complete timeline of her clinical case is presented in Fig. 1, while Table 1 summarizes the main patient’s clinical and biochemical/imaging features.

**Table 1:** Main index patient’s clinical and biochemical/imaging features

Primary MEN1 tumor types	Main clinical manifestations	Other clinical features	Main biochemical features	Main imaging features
Bilateral parathyroid adenomas	Primary hyperparathyroidism	Anorexia	Elevated serum PTH levels	Parathyroid nodules at neck US and CT scans
		Bone fractures	Hypercalcemia	Parathyroid nodules at technetium-99m sestamibi scintigraphy
			Hypomagnesemia	Parathyroid nodules at 18-fluorocholine-PET-CT scan
			Elevated BALP	Pelvic fractures at X-ray scan
				Decreased BMD at CT and DEXA scans
				Increased bone remodeling at CT scan
				Post-parathyroidectomy hungry bone syndrome
Functioning pancreatic islet glucagonomas	Glucagonoma syndrome	Glucose intolerance/diabetes mellitus	Hyperglycemia	Pancreatic NETs at CT scan
	DVT	Weight loss	Elevated serum glucagon levels	Pancreatic NETs at MRI scan
		NME		Pancreatic NETs at PET/CT 68Ga-DOTATOC scan
		Portal vein thrombosis		DVT at CT scan
Non-functioning left adrenal adenoma	−	−	−	Adrenal mass at CT scan
				Adrenal mass at MRI scan
				Adrenal mass at PET/CT 68Ga-DOTATOC scan
Cutaneous collagenomas	Abdominal papules	−	−	−

*BALP* bone-specific alkaline phosphatase, *BMD* bone mineral density, *CT* computed tomography, *DEXA* dual-energy X-ray absorptiometry, *DVT* deep vein thrombosis, *MEN1* multiple endocrine neoplasia type 1, *MRI* nuclear magnetic resonance, *NETs* neuroendocrine tumors, *NME* necrolytic migratory erythema, *PET* positron emission tomography,* PTH* parathyroid hormone, *US* ultrasonography

The patient was the first-born of distantly related parents from Pakistan, after a full-term pregnancy. She had one sister and three younger brothers in good health; her mother died of kidney failure, and her father was living with heart disease and diabetes mellitus. She had already been hospitalized one year before and treated with bilateral parathyroidectomy after a diagnosis of primary hyperparathyroidism in parathyroid adenomas complicated by a pathological fracture of the pelvis and a post-intervention hungry bone syndrome (Online Resource 3); it is to note that the immunohistochemistry assay demonstrated a complete loss of menin expression in the tumoral tissue (Online Resource 4). The patient was then lost at follow-up and autonomously discontinued all therapies. Except for this hospitalization, her remote pathological history was unremarkable.

On admission, the patient underwent laboratory testing (Online Resource 5 panel a) and an ultrasound of the neck to detect possible residual parathyroid adenomas: the examination revealed a hypoechoic lesion at the right thyroid lobe of 20 × 12 × 13 mm and a isoechoic lesion to the left lobe of 10 mm. Technetium T-99 sestamibi parathyroid scintigraphy then revealed delayed washout of radiotracer near the upper pole of the right thyroid lobe and the lower pole of the left thyroid lobe corresponding to the lesions found on ultrasound; such findings, being not detectable at the thyroid scintigraphy after the administration of pertechnetate, were compatible with parathyroid hyperplasia or adenomas (Online Resource 6 panel a). Since the patient had already undergone subtotal parathyroidectomy the previous year and considering the current calcium levels only moderately elevated in the absence of new fractures, it was decided to treat the hypercalcemia only with medical therapy (initially, bisphosphonates).

Following the detection of hyperglycemia, a dosage of glycosylated hemoglobin [9.1%, 76.0 (NR 22.4–44.3) mmol/mol], and C-peptide [1.45 (NR 0.26–1.39) nmol/L] was performed. Glucagon dosage was also required, which resulted slightly elevated [252 (NR 25–250) ng/L]. Type 1 diabetes autoantibody panel, neuron-specific enolase (NSE), and chromogranin resulted within normal limits. A diagnosis of secondary decompensated diabetes was then made, and the patient started basal-bolus insulin therapy.

On suspicion of pancreatic glucagonoma, the patient underwent a computed tomography (CT) (Online Resource 6 panels b–) and subsequently a nuclear magnetic resonance (MRI) of the abdomen (Online Resource panels 7 panels a–d), which both disclosed multiple pancreatic masses (mainly at the tail) with maximum diameter of 16 mm and a left adrenal mass of 22 mm. Both imaging methods could not definitively rule out the possible malignancy of the latter lesion. Carcinoembryonic antigen and carbohydrate antigen 19-9 levels were within reference ranges. The exocrine function of her pancreas and gastrin levels was also normal, as well as gastroscopy.

To better characterize the newly identified lesions, a positron emission tomography (PET)/CT with 68 Ga-DOTATOC was performed, which showed multiple intense 68 Ga-avid areas in the pancreas head, body and tail, confirming the presence of pancreatic neuroendocrine tumors (Online Resource 8). Due to the physiological uptake of the tracer at the adrenal level, again it was not possible to fully characterize the left nodularity reported on CT and MRI. To exclude secreting adrenal adenomas, a dosage of 17-β-estradiol, testosterone, dehydroepiandrosterone sulfate, urinary cortisol, and urinary metanephrines was performed, all resulting within normal limits. Following high clinical suspicion for MEN1, pituitary gland was evaluated with a laboratory workup and a targeted MRI imaging that excluded pituitary hypersecretion and/or adenomas.

Physical examination revealed multiple smooth skin-colored papules of 0.5 cm diameter all over the abdomen. The patient reported having these cutaneous lesions from many years. A skin biopsy was performed, and the histological examination was compatible with skin collagenomas, a typical non-endocrine manifestation in MEN1 (Online Resource 7 panels e–f).

The patient was then evaluated by surgeons and finally performed a total pancreatectomy, splenectomy, and left adrenalectomy. Pancreas histological examination confirmed multiple neuroendocrine well-differentiated G1 tumors (Ki-67 proliferation index < 3%; absence of necrosis or vascular embolization; occasional foci of perineural invasion; staging: pT1N0M0); immunohistochemical staining of the neoplastic cells was strongly positive for glucagon, but negative for menin. Left adrenal gland was confirmed as a benign well-differentiated adrenal adenoma (Weiss score: 0): again, a complete loss of menin expression was found at immunohistochemistry (Online Resource 9). Main post-operation laboratory examinations are shown in Online Resource 5 panel b; thrombocytosis is attributable to previous splenectomy. It is to note that the intervention was early complicated by portal vein thrombosis.

Subsequently for about two years after the second surgery, the patient has been followed up in an endocrinology outpatient clinic: her performance status gradually improved, even if some issues remain unresolved (last follow-up blood tests summarized in Online Resource 5 panel c). Briefly, no new tumor masses have since appeared. Her diabetes mellitus shows persistent poor glycemic control during basal-bolus insulin therapy, despite the fact she is currently on Flash Glucose Monitoring (FGM) which so far has been preferred to an insulin pump because of her persistent language barrier and inability to manage the device. Moreover, during her post-operatory follow-up, a skin rash appeared at the left leg, which was diagnosed as a likely necrolytic migratory erythema (NME). Finally, for what concerns her relapsed hyperparathyroidism (last 18F-fluorocholine PET shown in Online Resource 10), persistent hypercalcemia began to be better controlled since starting calcimimetic therapy (last dosage of total calcium: 2.65 mmol/L). However, the surgical intervention has been so far delayed first due to SARS-CoV-2 pandemic and then because the patient’s family moved abroad (to the UK), where hopefully it will finally be performed in the next future.

### Results of genetic tests in the index patient and in her progeny

**Fig. 2:**
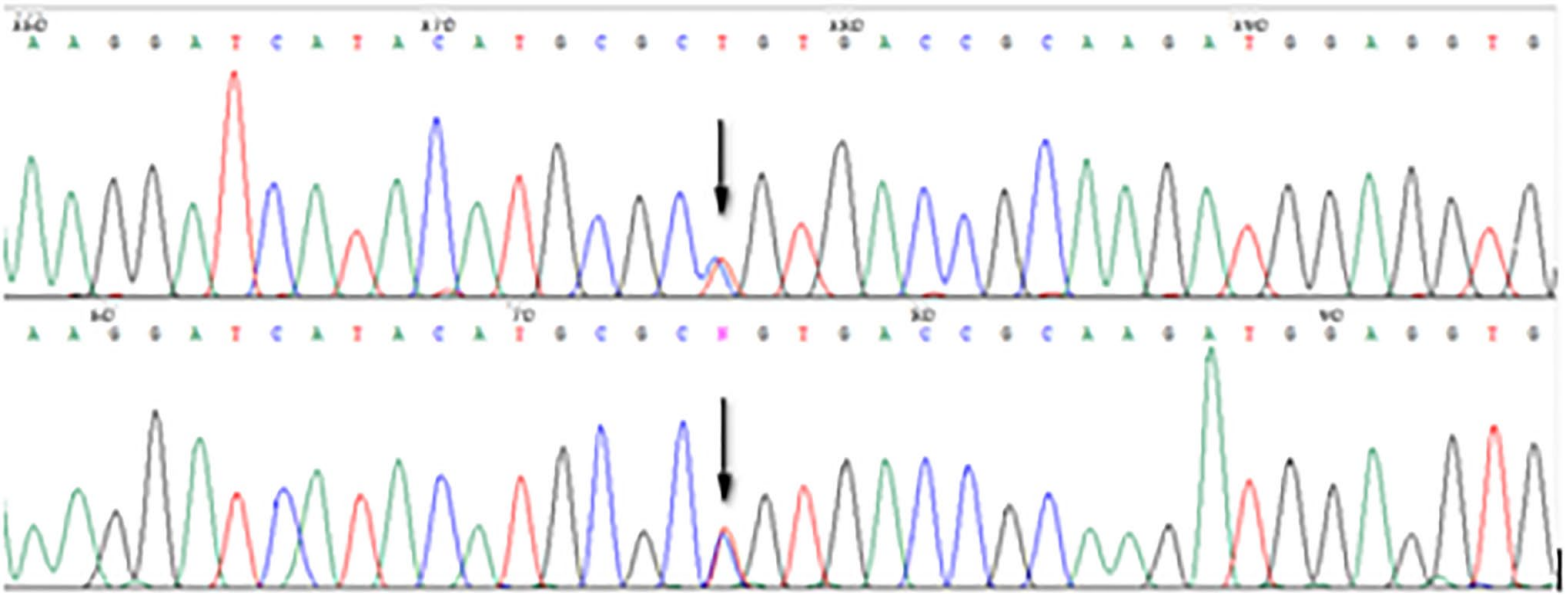
Reverse and forward Sanger sequencing, confirming on a second DNA extraction from peripheral blood the c.703T > C nucleotide substitution (indicated by the arrow) in *MEN1* exon 4 (NM_000244.3) identified by next-generation sequencing

Patient’s NGS resulted in a mean coverage of the target regions of 1234 reads (at least 50 reads for the entire target). No pathogenic variants (including CNVs) were found in the coding region of the 24 genes predisposing to endocrine tumors. However, a new variant of *MEN1* gene (c.703T > C, p.Cys235Arg NM_000244.4, rs1555165570) was found in heterozygosity (Fig. 2).

A new genetic counseling was subsequently carried out. Taking into account that the transmission of MEN1 is autosomal dominant and each affected subject has a risk of 50% to transmit it on to offspring, the geneticist suggested to search the same variant also in her two children. Only the 11-year-old daughter was found to have in heterozygosity the same nucleotide substitution in *MEN1* gene. She was then referred to pediatric endocrinology outpatient clinic and offered a screening test for MEN1-related diseases, but the mother refused because the family was in the meantime moving again abroad.

As it is currently unknown if the index patient’s variant was de novo or inherited, she was recommended to inform her first-degree family members (residing in Pakistan), who may have inherited the nucleotide substitution and will benefit from a genetic counseling.

### Clinical results in the patient's progeny

Both the patient’s daughter and the son were (and still are) asymptomatic and with unremarkable physical examination. The main clinical findings of the girl carrying the variant of interest are better detailed in Online Resource 11.

### In silico analysis of protein stability changes upon c.703T > C variant and prediction of the biological effect

The c.703T > C variant was identified within exon 4 of the *MEN1* gene, causing the substitution of the highly conserved cysteine 235 with an arginine p.(Cys235Arg), also known as p.Cys230Arg on the short isoform with respect to the MANE (Matched Annotation from NCBI and EMBL-EBI) transcript NM_001370259.2:c.688T > C). This variant is absent in the literature and in the main databases of human genome variability (1000 genomes phase 3, ESP, gnomAD), although ClinVar hosts a single entry suggesting a likely pathogenic effect (GeneDx, December 5, 2018; accession #RCV000519935.2).

**Fig. 3:**
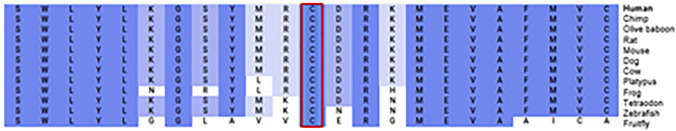
Sequence alignment of menin protein across 12 species (human amino acids from 224 to 246). Cysteine 235 is within the red rectangle

**Fig. 4:**
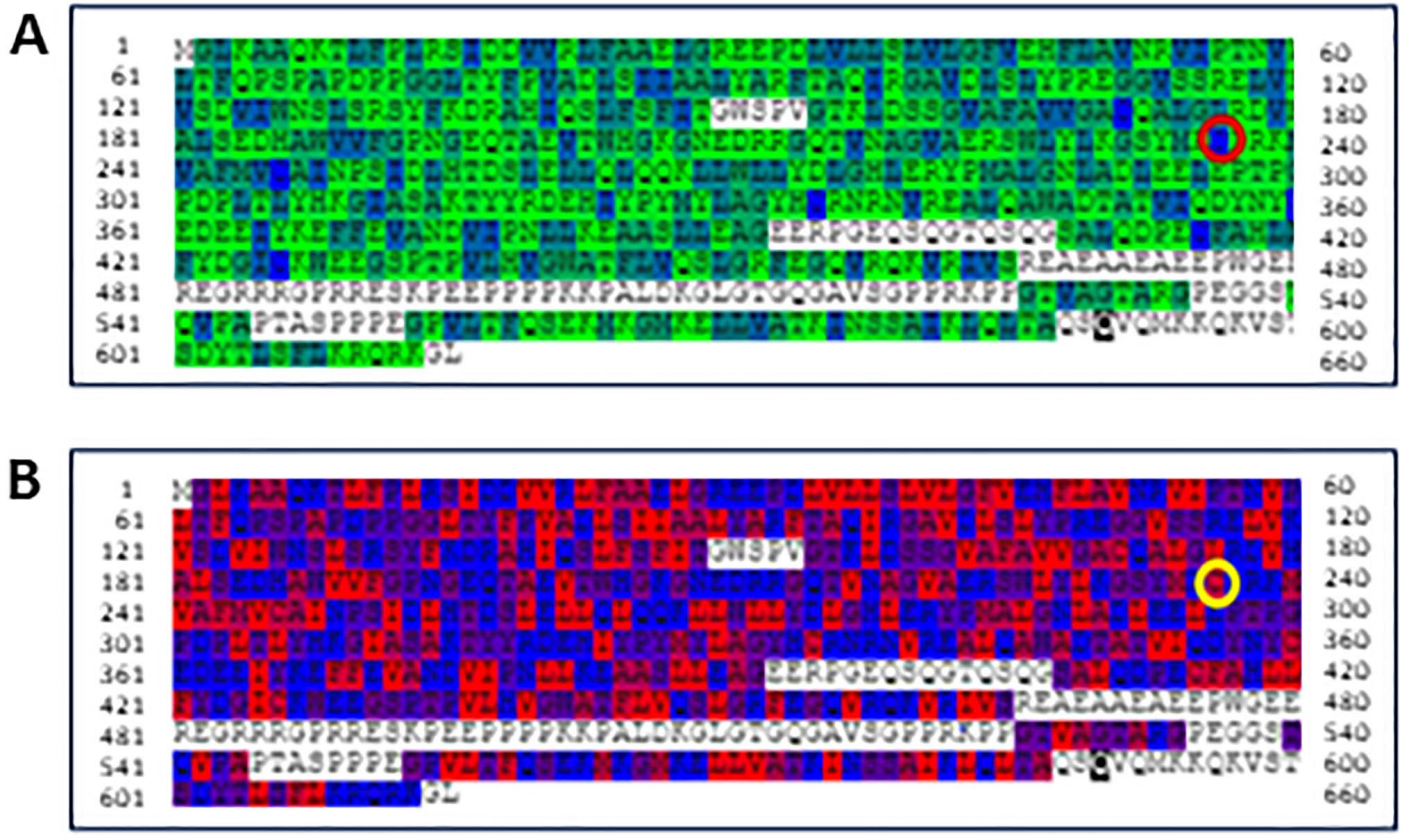
Analysis of the structural impact of the missense variant recognizes for cysteine 235 of menin (sequence browser: O00255-1) highest buried and hydrophobicity indices suggesting a deleterious effect for p.Cys235Arg substitution (Missense3D)

**Fig. 5:**
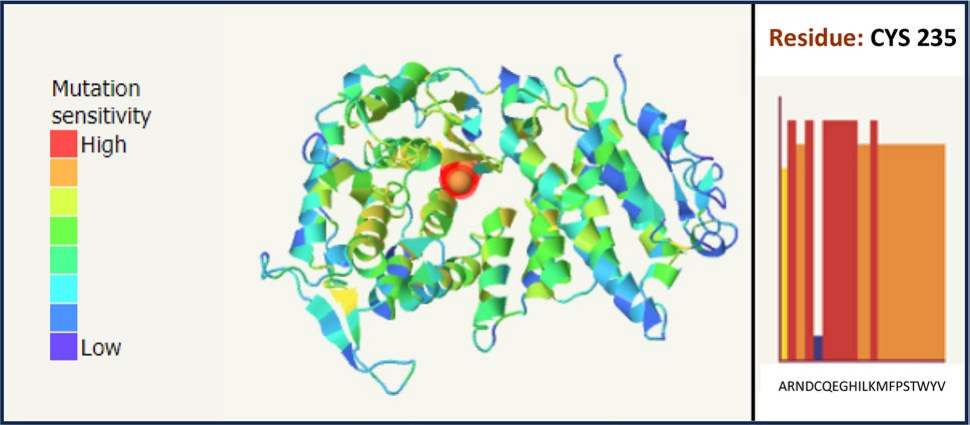
Crystallographic structure of the menin protein showing an "internal" (buried) position for the hydrophobic cysteine 235 and a high score of intolerability toward any amino acid substitution in this protein position with particular regard to hydrophilic and charged residues such as a basic arginine. The higher and red the bars are, the more the amino acid substitution is predicted not tolerated (Missense3D)

**Fig. 6:**
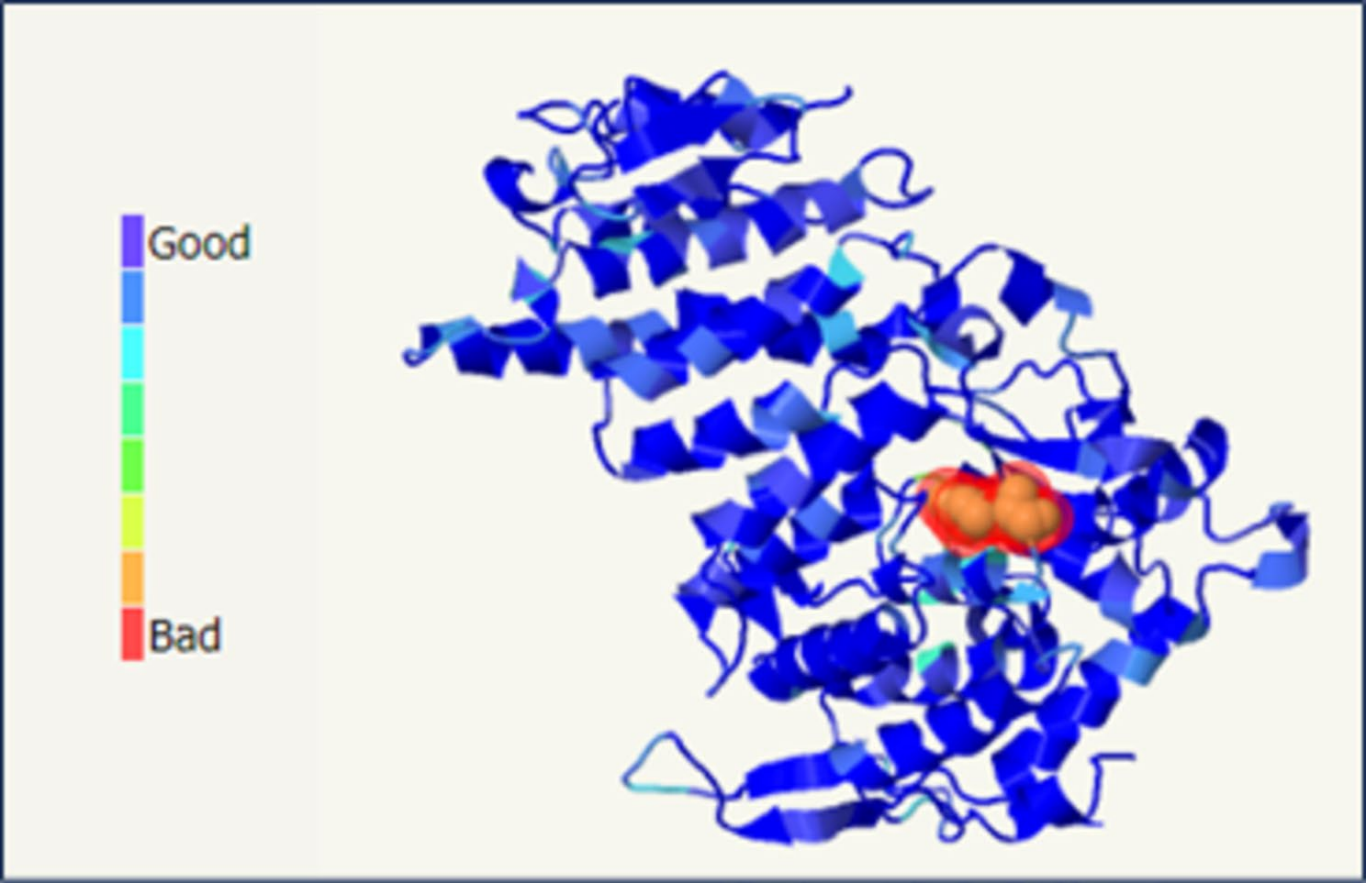
Steric clash analysis of the menin protein with arginine at position 235, instead of cysteine, suggests a high rate of clashes that means bad side-chain placement, or possibly an incorrect backbone in the region (Phyre2)

While the nucleotide substitution does not seem to interfere with the splicing of the exon 4, it causes a non-conservative amino acid change between the hydrophobic cysteine and the basic/hydrophilic arginine (Grantham Distance: 180—BLOSUM62: -3): according to Uniprot database, it involves a residue that is almost invariant in evolution (Fig. 3), within the domain of interaction with the FA complementation group D2 (FANCD2) protein. In particular, the cys235 residue is located at the border between the beta-sheet region 6 and the alpha-helix 9. Missense3D analysis of the structural impact of the missense variant recognized for menin cysteine 235 highest buried and hydrophobicity indices, suggesting a deleterious effect for p.Cys235Arg substitution (Fig. 4). Moreover, the crystallographic structure of the menin protein showed an “internal” (buried) position for the hydrophobic cysteine 235 and a high score of intolerabilities toward any amino acid substitution in this protein position with particular regard to hydrophilic and charged residues such as a basic arginine (Missense3D, Phyre2) (Fig. 5). Finally, clash analysis predicted a bad side chain for the newly introduced Arg235 and, possibly, an incorrect backbone of the region (Phyre2) (Fig. 6). Accordingly, twenty-two additional informatics tools agreed in predicting a deleterious effect of this variant with just two models returning an uncertain effect and none a benign effect (Online Resource 2) [16, 17]. Of note, seven predictors with further weighted scores gave a strong prediction toward pathogenicity.

In the absence of a functional test, we cannot assess whether the substitution interferes with the protein stability or with more subtle functions such as the binding with partner proteins. In any case, the concomitant evidence of loss of menin expression at the immunohistochemistry assay (Online Resource 4 and Online Resource 9) on different tumor specimens from the index case strongly supports the hypothesis of an early degradation of the instable variant protein coupled with a loss-of-function second hit occurring in the wild-type allele. In summary, the evidence collected so far suggested a likely pathogenetic biological effect (class 4, according to ACMG criteria as detailed in Online Resource 12 [15]).

## Discussion

Menin, the product of the *MEN1* gene, is a 67 kDa tumor suppressor protein whose mechanism of action is not completely understood. While *MEN1* mutations are only tumorigenic within neuroendocrine lineages, menin is expressed in most tissues at all stages of development and has probably a universal function. Germline mutations of *MEN1* gene result in MEN1 syndrome, a rare autosomal dominant predisposition to benign and malignant tumors.

The optimal role of genetic testing in the context of MEN1 is not clear cut, as there is a lack of high-quality data to demonstrate that preclinical detection of MEN1-related tumors leads to interventions that improve morbidity or mortality. However, the increased availability and application of genetic testing in clinical settings and the declining cost of DNA sequencing have removed some of the previous barriers to such procedures. Although the decision to undertake genetic testing should be carefully considered and fully discussed with the patient, current guidelines generally recommend it in patients considered to be at high risk of MEN1 [1]. Precisely regarding genetic testing for MEN1, in this research, we identified a unique rare missense single-nucleotide variant within exon 4 of the *MEN1* gene (cytogenetic location: 11q13.1) denoted NM_000244.3(MEN1):c.703T > C at the cDNA level, NP_000235.3:p.Cys235Arg at the protein level (Fig. 2). This variant results in the change of a cysteine to an arginine, and was not observed at significant frequency in large population cohorts. To the best of our knowledge, this variant, also known as c.798T > C or p.C235R, has not been published in the literature as a pathogenic or benign germline variant. ClinVar does not report functional evidence for this variant, although it hosts a single 2018 entry reporting clinical rationale that this is a germline mutation with a likely pathogenic effect (accession # RCV000519935.2), as confirmed in the last revision on February 2023 (accession # RCV000519935.7); no further data have so far been released concerning, for instance, the number of tested individuals or affected family members if any, their family history or the complete list of tested chromosomes [18, 19].

What is already known is that the variant is located in the region of specific menin interaction with FANCD2 (UniProt), in turn a protein encoded by a gene involved in DNA repair and mutated in patients with an inherited cancer-prone syndrome, Fanconi anemia. Since menin is predominantly localized in chromatin and nuclear matrix and its loss of expression in mouse embryonic fibroblasts has been associated to increased sensitivity to DNA damage, it is likely that it plays a role in DNA damage repair in concert with FANCD2 [20].

In the present study, we confirmed and expanded previous evidence, demonstrating for the first time that the aforementioned variant was present not only in the index patient, but also in the progeny. Moreover, we conducted several in silico analyses, which included evolutionary conservation patterns and their influence on identifying protein functional sites. All tested models supported a deleterious effect of this variation on both protein structure and function, in the sense that replacing cys235 with a basic (hydrophilic), arginine was predicted to introduce a charge in a protein position which is intolerant to any substitution (Figs. 4, 5), causing severe steric clashes (Fig. 6). Taken together, our data added additional information to the previously cited report that preliminary suggested p.Cys235Arg as a new likely pathogenic variant. More in detail, this definition applies when available data do not allow to correlate with absolute certainty a genetic variant with a target disease. In this case, the variant has not been described in other patients, with the exception of what above reported from a single submitter, but its characteristics—together with the high specificity of the patient’s clinical phenotype—pointed toward a possible causative role (Online Resource 12) [15]. As far as our index patient was concerned, *MEN1* mutational analysis did not possibly result in a diagnostic power gain—since there were already largely adequate clinical criteria to support the diagnosis, all the more so in a case like this with multiple manifestations of disease as will discussed hereafter—but it was useful in other linked ways, such helping to establish whether mutation/variant-specific carrier testing could be offered to relatives in that family or definitively determining whether or not asymptomatic or other relatives of the proband carried the same genetic variant (as was the case of the patient’s daughter).

In our opinion, our research presents insights of interest also from a more purely clinical point of view. As a matter of fact, the index patient fulfilled the main clinical diagnostic criteria for MEN1, being affected by three of the primary endocrine tumors which have been associated with this syndrome (i.e., the classic parathyroid and adrenal gland adenomas, in addition to the extremely rare pancreatic glucagonomas). Furthermore, the subject presented multiple cutaneous collagenomas from many years, which have 50–65% sensitivity and 92–100% specificity for MEN1, but with increased diagnostic performance in combination with pancreatic endocrine tumors [21]. In our opinion, of all these clinical manifestations, the most interesting one is undoubtedly the presence of glucagonomas. These are very uncommon tumors that originate almost exclusively in the pancreas (from α-2 islet cells), with an annual incidence of 0.05–0.1/1,000,000 (around 1% of all neuroendocrine tumors) [22–25]. They are characterized by the so-called glucagonoma syndrome, i.e., glucagon overproduction, weight loss, hyperglycemia, diabetes mellitus, hypoaminoacidemia, normochromic normocytic anemia, and necrolytic migratory erythema (NME) [26, 27]. NME is a very rare paraneoplastic dermatologic condition (estimated incidence of one per 20 million) presenting as phlogistic damage to tissues in areas exposed to friction and pressure, and representing the most characteristic clinical sign of this condition [28]. It is to note that the index patient had also portal vein thrombosis, possibly also attributable to glucagonoma syndrome. Indeed, venous thrombosis is thought to occur in as many as 30% of these subjects; conversely, patients with an unexplained thromboembolic disease should undergo a thorough history, physical and evaluation for the possibility of glucagonoma [26, 27].

Glucagonomas generally develop as sporadic neoplasms [28–31], and only 20% are familial forms [32]. The latter ones are generally associated with MEN1—as in the case of our patient—or to the extremely rare Mahvash disease, a familial pancreatic α-cell hyperplasia and glucagonoma due to inactivating mutations in the glucagon receptor (*GCGR*) gene [33]. In any case, the available literature on familiar clusters is extremely limited. While familial autosomal dominant hyperglucagonemia has long been described [34], there is—to the best of our knowledge—only one report of hereditary glucagonomas from the group of Stacpoole. They described a family in which three persons had α-cell pancreatic tumors (i.e., glucagonomas) as part of MEN1, among which two had the classic glucagonoma syndrome with skin rash, glucose intolerance, and hypoaminoacidemia [35]. In any case, the paper did not analyze the possible underlying genetic factors. Thus, currently, a family history of MEN1 remains a recognized risk factor for this neoplasia, but information on the exact genetic mechanisms is quite poor [36–40]. Similarly, other data suggest that 17% of patients with seemingly sporadic pancreatic neuroendocrine tumors harbor germline alterations in any one of a variety of genes (including *MUTYH*, *CHEK2*, and *BRCA2*, as well as *MEN1* and *VHL*), suggesting that all patients with glucagonoma should at least be considered for inherited genetic syndromes testing (including, but not limited to, MEN1) [41].

Coming back to our index patient, she fulfilled the necessary criteria to diagnose a glucagonoma [42]: in fact, we demonstrated the presence of increased levels of blood glucagon in a patient with glucose intolerance (Online Resource 5), we found the tumor mass with direct visualization at both diagnostic (Online Resource 6 and Online Resource 7) and nuclear medicine imaging (Online Resource 8) and we finally obtained a histological confirmation (Online Resource 9).

As regards the carrier progeny, it should be underlined that she is too young for any speculation on the penetrance of the disease, given the diagnosis of MEN1 in the first two decades of life in only 15–17% of cases and an estimated penetrance of over 50% by the age of 20 and 95% by the age of 40 years [43].

## Conclusions

This study underscores the importance of early diagnosis and close follow-up in patients with MEN1. Any patient with clinical suspicion of MEN1 should be evaluated with appropriate biochemical screening tests, imaging of typically involved organs and evaluation by a multidisciplinary team including an endocrinologist.

Moreover, the research presented here stresses the importance of genetic testing when such diseases are suspected. Here, the genetic analysis performed in the index patient did not allow to identify any mutation of certain pathogenic effect or deletions/duplications in the coding regions of the main genes predisposing to the development of MEN1, MEN1-like or MEN4 (*MEN1*, *CDKN1B-p27*, *CDKN1A-p21*, *CDKN2B-p15*, *CDKN1C-p18*, *AIP*, *CDC73*). However, we found a novel variant (c.703T > C) in exon 4 of *MEN1* gene. The finding of the same substitution also in the progeny strengthened the possibility of a causative role for this genetic disorder and could contribute to its possible future reclassification as a “pathogenic variant” [44].

### Supplementary Information

Below is the link to the electronic supplementary material.

Supplementary file1 (PDF 50 KB)

Supplementary file2 (PDF 42 KB)

Supplementary file3 (PDF 96 KB)

Supplementary file4 (PDF 248 KB)

Supplementary file5 (PDF 129 KB)

Supplementary file6 (PDF 88 KB)

Supplementary file7 (PDF 115 KB)

Supplementary file8 (PDF 47 KB)

Supplementary file9 (PDF 375 KB)

Supplementary file10 (PDF 88 KB)

Supplementary file11 (PDF 148 KB)

Supplementary file12 (PDF 92 KB)

## Data Availability

All data generated or analyzed during this study are included in this published article and its supplementary information files.

## Code availability

No previously unreported custom computer codes or algorithms were used to generate results that are reported in the paper. Computer codes/softwares that were deemed central to the conclusions are reported in Online Resource 2, and have no restrictions to access.
