# Supplementary material for: A novel likely pathogenetic variant p.(Cys235Arg) of the MEN1 gene in multiple endocrine neoplasia type 1 with multifocal glucagonomas
Source: J Endocrinol Invest. 2024 Jan 31;47(7):1815–25. doi: 10.1007/s40618-023-02287-x (PMC11196359; doi:10.1007/s40618-023-02287-x)
Supplement: Supplementary file 1 — Supplementary file1 (PDF 50 KB) [file 40618_2023_2287_MOESM1_ESM.pdf]

Online Resource 2 List of matrices, *in silico* predictors and databases used for the current study and the score obtained for the *MEN1* variant c.703T>C (up-date November 2023).

Article title: A novel likely pathogenetic variant p.(Cys235Arg) of the *MEN1* gene in multiple endocrine neoplasia type 1 with multifocal glucagonomas

Journal name: Journal of Endocrinological Investigation

Author names: Carlo Smirne, Greta Maria Giacomini, Alessandro Maria Berton, Barbara Pasini, Francesca Mercalli, Flavia Prodam, Marina Caputo, Lodewijk Adriaan Anton Brosens, Edoardo Luigi Maria Mollero, Rosa Pitino, Mario Pirisi, Gianluca Aimaretti, Ezio Ghigo

Affiliation and e-mail address of the corresponding author: Department of Translational Medicine, University of Piemonte Orientale, 28100 Novara, Italy. Email: carlo.smirne@med.uniupo.it

Type of tool	Name	Website	Score
**Matrix**[1]	Grantham	https://gist.github.com/danielecook/501f03650bca6a3db31ff3af2d413d2a	180 (0 to 215)
	BLOSUM62	https://www.ncbi.nlm.nih.gov/Class/FieldGuide/BLOSUM62.txt	-3 (11 to -4)
**Predictor**[2]	CADD	https://cadd.gs.washington.edu/	Phred 26,8
	DANN	https://cbcl.ics.uci.edu/public_data/DANN/	0,99
	Align-GVGD	http://agvgd.hci.utah.edu/index.php	C65 (D, S)
	PolyPhen-2	http://genetics.bwh.harvard.edu/pph2/	HumVar 1 (D)<br>HumDiv 0,99 (D)
	MutationTaster	https://www.mutationtaster.org/	94 | 6 (D)
	SIFT	https://sift.bii.a-star.edu.sg/	0 (D, P)
	PROVEAN	http://provean.jcvi.org/index.php	-10,84 (D, P)
	BayesDel	https://fengbj-laboratory.org/BayesDel/BayesDel.html	noAF 0.5674 (D, S)<br>addAF 0.5606 (D, S)
	REVEL	https://sites.google.com/site/revelgenomics/	0.959 (D, S)
	MetaRNN	https://varsome.com/variant/hg38/NM_001370259.2%3Ac.688T%3EC?annotation-mode=germline	0.9916 (D, S)
	MetaLR	https://sites.google.com/site/jpopgen/dbNSFP	0.959 (D, P)
	DEOGEN2	http://deogen2.mutaframe.com/<br>https://bio.tools/DEOGEN2	0.9844 (D, S)
	MutPred2	http://mutpred.mutdb.org/	0.901 (D, S)
	FATHMM-MKL	https://fathmm.biocompute.org.uk/fathmmMKL.htm	0.9677 (D, M)
	M-CAP	http://bejerano.stanford.edu/mcap/	0.9335 (D, M)
	MVP	https://github.com/ShenLab/missense	0.9912 (D, M)
	EIGEN	http://www.columbia.edu/~ii2135/eigen.html	0.7171 (D, P)
	LRT	by Varsome	0 (D, P)
	PrimateAI	https://github.com/Illumina/PrimateAI	0.8331 (D, P)
	VARITY	https://sites.google.com/site/jpopgen/dbNSFP	0.98 (D)
	Franklin	https://franklin.genoox.com/clinical-db/home	Aggregated prediction 0.99 (D) Likely pathogenic
	Varsome	https://varsome.com/variant/hg38	Aggregated prediction 9 points (D) Likely pathogenic
	LIST-S2	https://list-s2.msl.ubc.ca/;jsessionid=686EBE2822265217A1638E5EF6B898F6?session=686EBE2822265217A1638E5EF6B898F6	0.8979 (U)
	MutationAssessor	http://mutationassessor.org/r3/	2.66 (U)
	Missense3D	http://missense3d.bc.ic.ac.uk/missense3d/	
	Phyre2	http://www.sbg.bio.ic.ac.uk/~phyre2/html/page.cgi?id=index	
**Database**	1000 genomes phase 3	https://www.internationalgenome.org/data-portal/data-collection/phase-3	

ESP	https://evs.gs.washington.edu/EVS/
gnomAD	https://gnomad.broadinstitute.org/
ClinVar	https://www.ncbi.nlm.nih.gov/clinvar/
Uniprot	https://www.uniprot.org/

[1]Scores range from the more tolerated to the less tolerated, [2]Score D for predicted deleterious effect [when available, the annotation is further divided in strong (S), moderate (M) or supportive (P) prediction], U for uncertain effect. None predictor gave a benign prediction.
