# Supplementary material for: A novel likely pathogenetic variant p.(Cys235Arg) of the MEN1 gene in multiple endocrine neoplasia type 1 with multifocal glucagonomas
Source: J Endocrinol Invest. 2024 Jan 31;47(7):1815–25. doi: 10.1007/s40618-023-02287-x (PMC11196359; doi:10.1007/s40618-023-02287-x)
Supplement: Supplementary file 2 — Supplementary file2 (PDF 42 KB) [file 40618_2023_2287_MOESM2_ESM.pdf]

Online Resource 3 Main laboratory findings before and after parathyroidectomy. Bold values are outside local laboratory normal ranges.

Article title: A novel likely pathogenetic variant p.(Cys235Arg) of the *MEN1* gene in multiple endocrine neoplasia type 1 with multifocal glucagonomas

Journal name: Journal of Endocrinological Investigation

Author names: Carlo Smirne, Greta Maria Giacomini, Alessandro Maria Berton, Barbara Pasini, Francesca Mercalli, Flavia Prodam, Marina Caputo, Lodewijk Adriaan Anton Brosens, Edoardo Luigi Maria Mollero, Rosa Pitino, Mario Pirisi, Gianluca Aimaretti, Ezio Ghigo

Affiliation and e-mail address of the corresponding author: Department of Translational Medicine, University of Piemonte Orientale, 28100 Novara, Italy. Email: carlo.smirne@med.uniupo.it

		**Before parathyroidectomy**	**During parathyroidectomy**	**After parathyroidectomy**	**Local laboratory NR**
Bone metabolism	PTH	**291.0**	/	**135.0**	6.5-39.0 pg/mL
	Intact PTH, rapid intra-operative basal assay	/	**906.0**	/	11.0-72.0 pg/mL
	Intact PTH at manipulation	/	**287.0**	/	
	Intact PTH 10 minutes after excision	/	**140.0**	/	
	Ca, total	**2.62**	/	2.17	2.15-2.50 mmol/L
	Ca, ionized	/	/	1.24	1.15-1.35 mmol/L
	P	**0.48**	/	**0.55**	0.87-1.45 mmol/L
	ALP	/	/	**2075**	70-290 U/L
	BALP	**>240,0**	/	/	3.0-19.0 µg/L
	25-hydroxy vitamin D	**10.1**	/	/	30.0-100.0 ng/mL
	Calcitonin	1.1	/	/	1.0-14.0 pg/mL
Renal function and Na/K	Cr	**28.3**	/	**37.1**	52.8-96.8 µmol/L
	eGFR	148	/	135	>90 mL/min/1.73m^2
	Na	141	/	139	134-146 mmol/L
	K	4.1	/	3.9	3.5-5.5 mmol/L
Other hormones	TSH	0.905	/	/	0.450-3.500 mIU/L
	PRL	231.4	/	/	59.4-619.0 mIU/L
	Gastrin	37.8	/	/	13.0-115.0 pg/mL
	Chromogranin	41.9	/	/	0.0-108.0 ng/mL
Metanephrines (urinary)	Normetanephrine	277.5	/	147,6	105.0-354.0 µg/24h
	Metanephrine	**327.0**	/	165,6	74.0-297.0 µg/24h
	3-methoxytyramine	**373.5**	/	138,6	55-247 µg/24h

ALP, total alkaline phosphatase; BALP, bone-specific alkaline phosphatase; Ca, calcium; Cr, creatinine; eGFR, estimated glomerular filtration rate; K, potassium; Na, sodium; NR, normal range; P, phosphorus; PRL, prolactin; PTH, parathyroid hormone; TSH, thyroid stimulating hormone; /, not tested.
