# Supplementary material for: A novel likely pathogenetic variant p.(Cys235Arg) of the MEN1 gene in multiple endocrine neoplasia type 1 with multifocal glucagonomas
Source: J Endocrinol Invest. 2024 Jan 31;47(7):1815–25. doi: 10.1007/s40618-023-02287-x (PMC11196359; doi:10.1007/s40618-023-02287-x)

Online Resource 4 Main findings regarding the patient's parathyroid adenomas. **a** 99Tc-sestamibi parathyroid scintigraphy with two focal uptakes (arrows), the larger in the left thyroid aspect and the smaller in the superior pole of the right thyroid lobe. **b** CT of the neck revealing an enlarged right parathyroid with significant contrast enhancement (arrow). **c** CT of the neck revealing significant contrast enhancement in the left parathyroid (arrow). **d** Histopathologic image of tissue right parathyroidectomy stained with hematoxylin and eosin (original magnification X 2) and consistent with parathyroid adenoma. **e** Another histopathologic image of right parathyroidectomy stained with hematoxylin and eosin (original magnification X 20). **f** Histopathologic image of tissue right parathyroidectomy stained for menin (original magnification X 20) and showing clear loss of menin in the tumor cells (right handside of the picture) with retained expression in internal controls, i.e. stromal cells like endothelial cells and fibroblasts (arrows) and in adjacent normal parathyroid tissue (green asterisk).

Article title: A novel likely pathogenetic variant p.(Cys235Arg) of the *MEN1* gene in multiple endocrine neoplasia type 1 with multifocal glucagonomas

Journal name: Journal of Endocrinological Investigation

Author names: Carlo Smirne, Greta Maria Giacomini, Alessandro Maria Berton, Barbara Pasini, Francesca Mercalli, Flavia Prodam, Marina Caputo, Lodewijk Adriaan Anton Brosens, Edoardo Luigi Maria Mollero, Rosa Pitino, Mario Pirisi, Gianluca Aimaretti, Ezio Ghigo

Affiliation and e-mail address of the corresponding author: Department of Translational Medicine, University of Piemonte Orientale, 28100 Novara, Italy. Email: carlo.smirne@med.uniupo.it

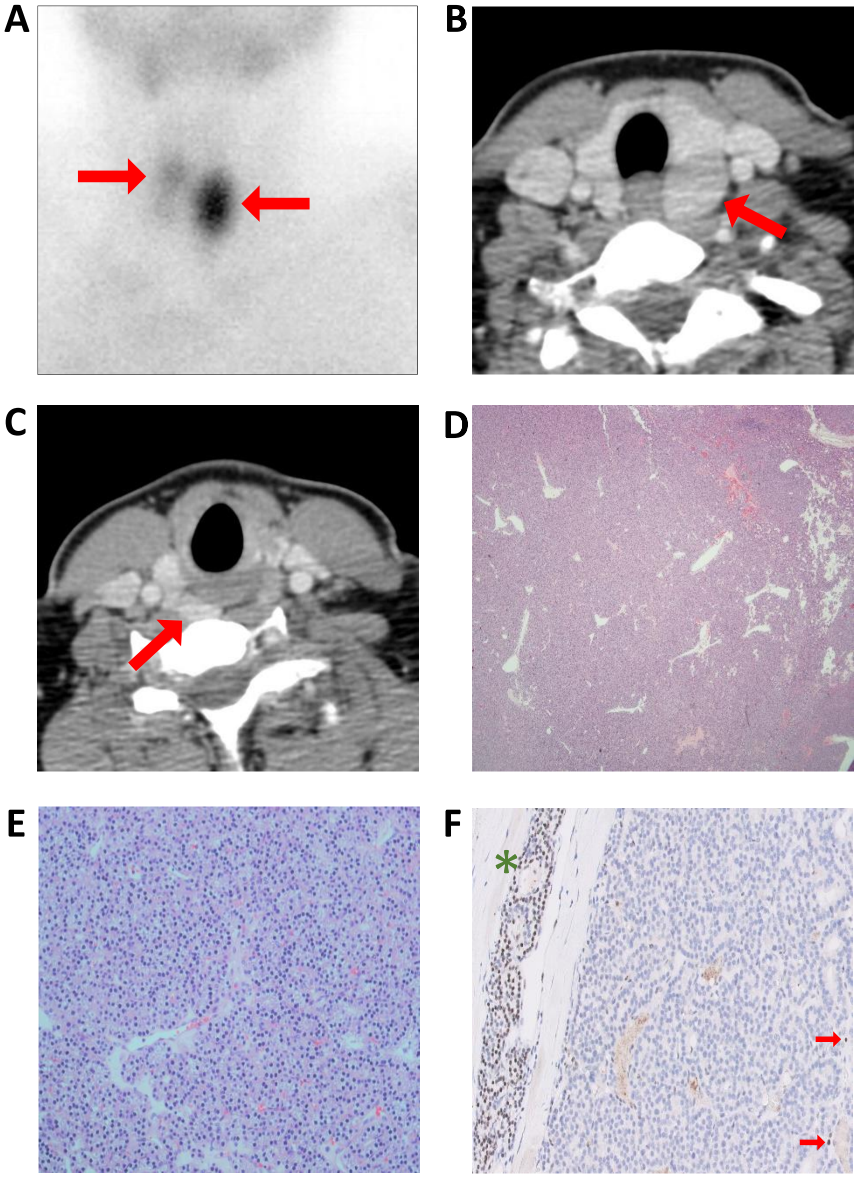

Supplement: Supplementary file 3 — Supplementary file3 (PDF 96 KB) [file 40618_2023_2287_MOESM3_ESM.pdf]
