# Supplementary material for: A novel likely pathogenetic variant p.(Cys235Arg) of the MEN1 gene in multiple endocrine neoplasia type 1 with multifocal glucagonomas
Source: J Endocrinol Invest. 2024 Jan 31;47(7):1815–25. doi: 10.1007/s40618-023-02287-x (PMC11196359; doi:10.1007/s40618-023-02287-x)
Supplement: Supplementary file 5 — Supplementary file5 (PDF 129 KB) [file 40618_2023_2287_MOESM5_ESM.pdf]

Online Resource 5 Main laboratory findings before and after pancreas and left adrenal surgical resection. Bold values are outside local laboratory normal ranges. **a** pre-operation **b** post-operation **c** last follow-up

Article title: A novel likely pathogenetic variant p.(Cys235Arg) of the *MEN1* gene in multiple endocrine neoplasia type 1 with multifocal glucagonomas

Journal name: Journal of Endocrinological Investigation

Author names: Carlo Smirne, Greta Maria Giacomini, Alessandro Maria Berton, Barbara Pasini, Francesca Mercalli, Flavia Prodam, Marina Caputo, Lodewijk Adriaan Anton Brosens, Edoardo Luigi Maria Mollero, Rosa Pitino, Mario Pirisi, Gianluca Aimaretti, Ezio Ghigo

Affiliation and e-mail address of the corresponding author: Department of Translational Medicine, University of Piemonte Orientale, 28100 Novara, Italy. Email: carlo.smirne@med.uniupo.it

		**A. Pre-operation**	**B. Post-operation**	**C. Last follow-up**	**Local laboratory NR**
Blood tests	WBC	6.38	8.28	10.07	4.50-11.00 x10^9/L
	RBC	4.79	4.53	4.56	3.80-5.20 x10^{12}/L
	Hb	131	144	141	117-157 g/L
	PLT	265	433	418	150-450 x10^9/L
Liver function	AST	32	**167**	76	0-40 U/L
	ALT	**91**	**112**	70	0-40 U/L
	ALP	**380**	**347**	220	70-290 U/L
	Albumin	45	41	41	34-48 g/L
Renal function and Na/K	Cr	**41.4**	**35.2**	**42.4**	52.8-96.8 µmol/L
	eGFR	129	136	127	>90 mL/min/1.73m^2
	UA	279.6	267.7	339.0	154.6-356.9 µmol/L
	Na	**133**	139	138	134-146 mmol/L
	K	**3.4**	4.5	4.5	3.5-5.5 mmol/L
Glucose metabolism	FPG	**14.3**	**8.3**	**10.5**	3.9-5.6 mmol/L
	HbA1c	**76.0**	**107.0**	**75.0**	22.4-44.3 mmol/mol
	C-peptide	**1.45**	/	/	0.26-1.39 nmol/L
	Glucagon	**252**	/	/	25-250 ng/L
Bone metabolism	Ca, total	**2.59**	**2.74**	**2.57**	2.15-2.50 mmol/L
	Ca, ionized	1.21	**1.39**	1.21	1.15-1.35 mmol/L
	P	**0.58**	1.03	1.07	0.87-1.45 mmol/L
	PTH	**139.0**	**85.6**	**75.0**	6.5-39.0 pg/mL
	25-hydroxy vitamin D	**9.7**	**7.2**	**16.6**	30.0-100.0 ng/mL
	BALP	/	**65.1**	/	3.0-19.0 µg/L
	Calcitonin	/	<1.0	/	1.0-14.0 pg/mL
Sex hormones	FSH	5.3	3.6	/	1.5-33.4 mIU/mL
	LH	9.80	**1.60**	/	1.9-77.0 mIU/mL
	PRL	170.1	544.8	/	59.4-619.0 mIU/L
	PRG	/	<0.15	/	0.15-28.3 ng/mL
	17 β-Estradiol	/	29.0	/	18.9-256 pg/mL
	Testosterone	0.278	/	/	0.126-0.590 ng/mL
	DHEA-S	**0.169**	/	/	0.587-2.270 µg/mL
Cortisol	Urinary cortisol	**2.9**	/	/	3.5-45.0 µg/24h
	Urinary cortisone	**10.7**	/	/	17-129 µg/24h
	8 a.m. cortisol	/	**31**	/	a.m. 45-240 µg/L
	Cortisol during ACTH stimulation test at 0/30/60 minutes	/	51/176/220	/	basal a.m. 45-240 µg/L
Catecholamines (urinary)	Noradrenaline	19.7	/	/	15.0-86.0 µg/24h
	Adrenaline	5.4	/	/	2.0-25.0 µg/24h
	Dopamine	**207.4**	/	/	250.0-500.0 µg/24h
Metanephrines (urinary)	Normetanephrine	176.8	/	/	105.0-354.0 µg/24h
	Metanephrine	198.9	/	/	74.0-297.0 µg/24h
	3-methoxytyramine	122.4	/	/	55-247 µg/24h
Other hormones	NSE	8.39	/	10.10	0.00-12.50 ng/mL
	Chromogranin	34.7	24,2	65.9	0.0-108.0 ng/mL
	Gastrin	49.0	/	/	13.0-115.0 pg/mL
	IGF-1	/	178.7	/	107.8-246.7 ng/mL
	TSH	1.054	1.406	/	0.450-3.500 mIU/mL

ACTH, adrenocorticotrophic hormone; ALP, total alkaline phosphatase; ALT, alanine transaminase; AST, aspartate transaminase; BALP, bone-specific alkaline phosphatase; Ca, calcium; Cr, creatinine; DHEA-S, dehydroepiandrosterone sulfate; eGFR, estimated glomerular filtration rate; FPG, fasting plasma glucose; FSH, follicular stimulating hormone; Hb, hemoglobin; HbA1c, hemoglobin A1c; IGF-1, insulin-like growth factor 1; K, potassium; LH, luteinizing hormone; Na, sodium; NR, normal range; NSE, neuron-specific enolase: P, phosphorus; PLT, platelets; PRL, prolactin; PRG,

progesterone; PTH, parathyroid hormone; RBC, red blood cells; TSH, thyroid stimulating hormone; UA, uric acid; WBC, white blood cell; /, not tested.
