# Supplementary material for: A novel likely pathogenetic variant p.(Cys235Arg) of the MEN1 gene in multiple endocrine neoplasia type 1 with multifocal glucagonomas
Source: J Endocrinol Invest. 2024 Jan 31;47(7):1815–25. doi: 10.1007/s40618-023-02287-x (PMC11196359; doi:10.1007/s40618-023-02287-x)

Online Resource 7 Other main findings before pancreas and left adrenal surgical resection (part 2). a Abdomen contrast-enhanced MRI confirming a pseudonodular mass in the pancreatic tail with significant contrast enhancement, arterial phase (arrow). **b** The same pseudonodular mass in the pancreatic tail showed by contrast-enhanced MRI, portal venous phase (arrow). **c** A pseudonodular mass in the pancreatic body showed by T2-weighted contrast-enhanced MRI (arrow) **d** Left adrenal mass showed by T1-weighted contrast-enhanced MRI, arterial phase (arrow). **e** Histopathologic examination of skin biopsy stained with hematoxylin and eosin (original magnification X 2) and showing a polypid profile with thickened bands of collagen in the reticular dermis and loss of the boundary between papillary and reticular dermis, consistent with a collagenome. **f** A detail of the previous skin histopathologic image (original magnification X 10).

Article title: A novel likely pathogenetic variant p.(Cys235Arg) of the *MEN1* gene in multiple endocrine neoplasia type 1 with multifocal glucagonomas

Journal name: Journal of Endocrinological Investigation

Author names: Carlo Smirne, Greta Maria Giacomini, Alessandro Maria Berton, Barbara Pasini, Francesca Mercalli, Flavia Prodam, Marina Caputo, Lodewijk Adriaan Anton Brosens, Edoardo Luigi Maria Mollero, Rosa Pitino, Mario Pirisi, Gianluca Aimaretti, Ezio Ghigo

Affiliation and e-mail address of the corresponding author: Department of Translational Medicine, University of Piemonte Orientale, 28100 Novara, Italy. Email: carlo.smirne@med.uniupo.it

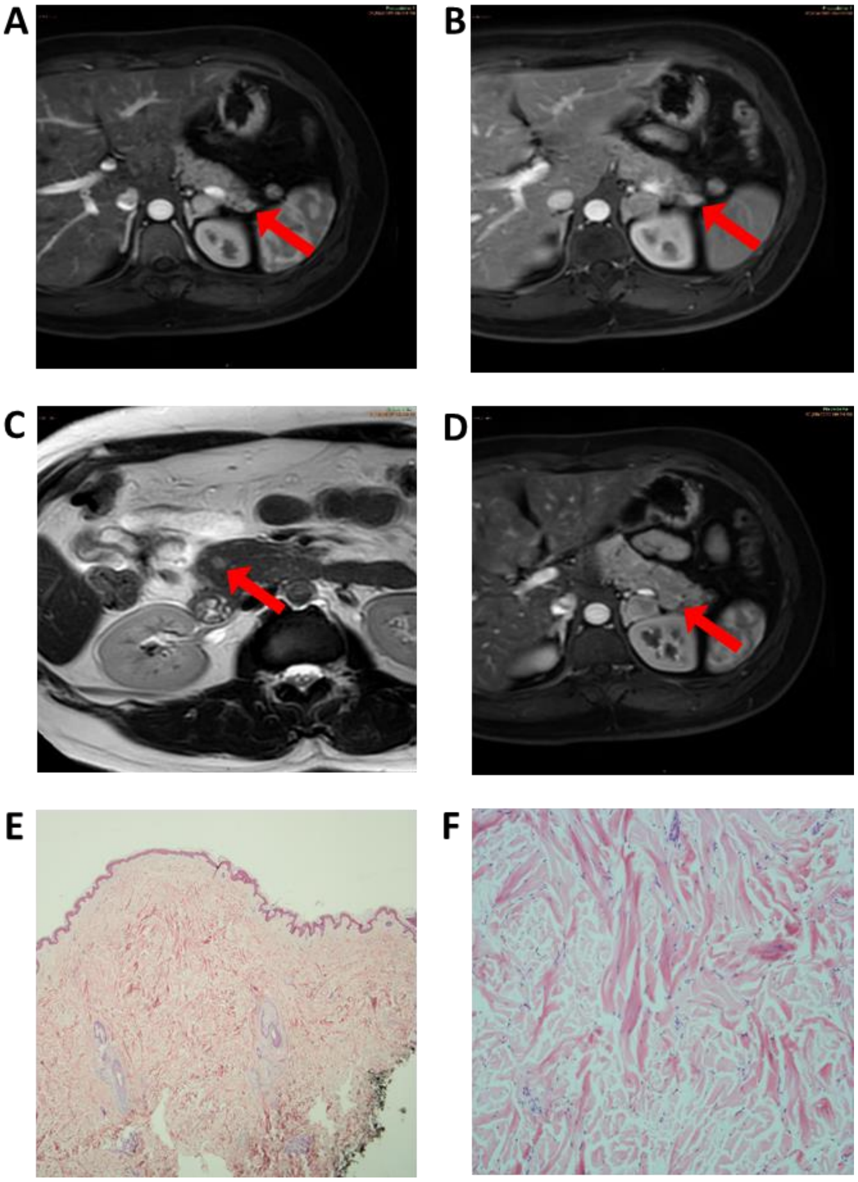

Supplement: Supplementary file 6 — Supplementary file6 (PDF 88 KB) [file 40618_2023_2287_MOESM6_ESM.pdf]
