# Supplementary material for: A novel likely pathogenetic variant p.(Cys235Arg) of the MEN1 gene in multiple endocrine neoplasia type 1 with multifocal glucagonomas
Source: J Endocrinol Invest. 2024 Jan 31;47(7):1815–25. doi: 10.1007/s40618-023-02287-x (PMC11196359; doi:10.1007/s40618-023-02287-x)

**Online Resource 8 Other main findings before pancreas and left adrenal surgical resection (part 3).** 68Ga-DOTATOC PET/CT showing multiple areas of increased uptake (arrows) both in pancreas body and tail, consistent with the pancreatic masses detected by CT and MRI (**a**-**b**). On the right handside are reported the corresponding transaxial CT images

**Article title:** A novel likely pathogenetic variant p.(Cys235Arg) of the *MEN1* gene in multiple endocrine neoplasia type 1 with multifocal glucagonomas

**Journal name:** Journal of Endocrinological Investigation

**Author names:** Carlo Smirne, Greta Maria Giacomini, Alessandro Maria Berton, Barbara Pasini, Francesca Mercalli, Flavia Prodam, Marina Caputo, Lodewijk Adriaan Anton Brosens, Edoardo Luigi Maria Mollero, Rosa Pitino, Mario Pirisi, Gianluca Aimaretti, Ezio Ghigo

**Affiliation and e-mail address of the corresponding author:** Department of Translational Medicine, University of Piemonte Orientale, 28100 Novara, Italy. Email: carlo.smirne@med.uniupo.it

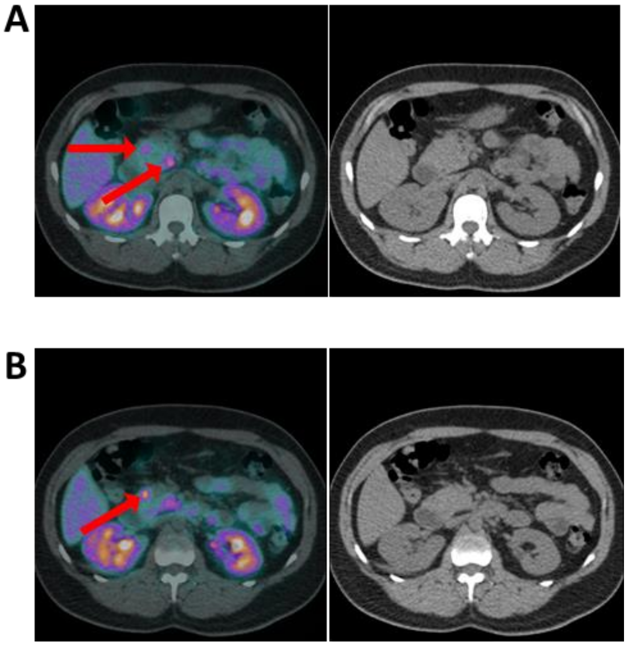

Supplement: Supplementary file 7 — Supplementary file7 (PDF 115 KB) [file 40618_2023_2287_MOESM7_ESM.pdf]
