# Supplementary material for: A novel likely pathogenetic variant p.(Cys235Arg) of the MEN1 gene in multiple endocrine neoplasia type 1 with multifocal glucagonomas
Source: J Endocrinol Invest. 2024 Jan 31;47(7):1815–25. doi: 10.1007/s40618-023-02287-x (PMC11196359; doi:10.1007/s40618-023-02287-x)

Online Resource 9 Pancreas histological examination confirming multiple neuroendocrine well-differentiated G1 tumors; left adrenal gland histological examination confirming a benign well-differentiated adenoma. Panels a-h refer to pancreatic cancer tissue except panel c which is normal pancreatic tissue. Panels i and j refer to adrenal tissue. **a** Immunohistochemical staining with hematoxylin and eosin (original magnification X 20). **b** Immunohistochemical staining for menin (original magnification X 20) showing clear loss of menin in the tumor cells with only very focally expression in a normal preexisting islet of Langerhans (red arrow) and in ducts (black arrow). **c** Immunohistochemical staining for glucagon (original magnification X 10) in normal pancreatic tissue with sparse endocrine islets. **d** Immunohistochemical staining for glucagon (original magnification X 20) in pancreatic tumor. **e** Immunohistochemical staining for chromogranin (original magnification X 20). **f** Immunohistochemical staining for Ki-67 (original magnification X 20). **g** Immunohistochemical staining for synaptophysin (original magnification X 20). **h** Immunohistochemical staining with hematoxylin and eosin showing focal perineural invasion (original magnification X 40). **i** Immunohistochemical staining with hematoxylin and eosin (original magnification X 20). **j** Immunohistochemical staining for menin (original magnification X 20) showing loss of menin in the tumor (red asterisk), with retained weak positivity in stromal cells (i.e. endothelial cells and fibroblasts) between the tumor cells and in adjacent preexisting normal adrenal tissue (green asterisk).

Article title: A novel likely pathogenetic variant p.(Cys235Arg) of the *MEN1* gene in multiple endocrine neoplasia type 1 with multifocal glucagonomas

Journal name: Journal of Endocrinological Investigation

Author names: Carlo Smirne, Greta Maria Giacomini, Alessandro Maria Berton, Barbara Pasini, Francesca Mercalli, Flavia Prodam, Marina Caputo, Lodewijk Adriaan Anton Brosens, Edoardo Luigi Maria Mollero, Rosa Pitino, Mario Pirisi, Gianluca Aimaretti, Ezio Ghigo

Affiliation and e-mail address of the corresponding author: Department of Translational Medicine, University of Piemonte Orientale, 28100 Novara, Italy. Email: carlo.smirne@med.uniupo.it

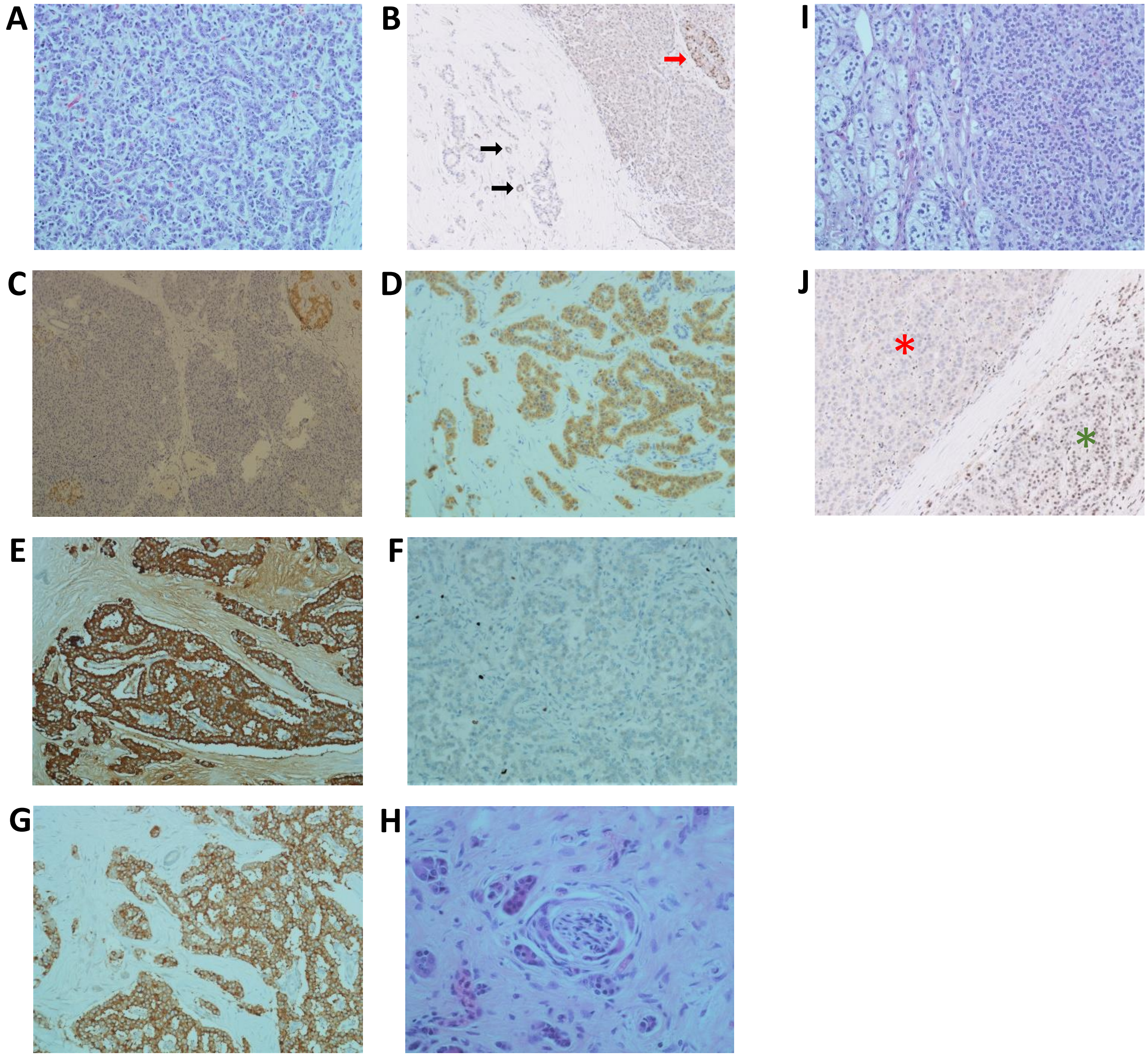

Supplement: Supplementary file 8 — Supplementary file8 (PDF 47 KB) [file 40618_2023_2287_MOESM8_ESM.pdf]
