# Supplementary material for: A novel likely pathogenetic variant p.(Cys235Arg) of the MEN1 gene in multiple endocrine neoplasia type 1 with multifocal glucagonomas
Source: J Endocrinol Invest. 2024 Jan 31;47(7):1815–25. doi: 10.1007/s40618-023-02287-x (PMC11196359; doi:10.1007/s40618-023-02287-x)

Online Resource 10 18^{F}-fluorocholine PET/CT for evaluation of parathyroids. a PET maximum-intensity projection (MIP) shows right posterior paratracheal uptake (arrows) consistent with the clinical suspicion of hyperplastic parathyroid with high phospholipid activity. **b-c** Corresponding transaxial CT and PET/CT images show a hypodense soft tissue lesion of about 1.2 × 0.8 x 1.2 cm with increased tracer uptake (arrows) suggesting of a parathyroid adenoma.

Article title: A novel likely pathogenetic variant p.(Cys235Arg) of the *MEN1* gene in multiple endocrine neoplasia type 1 with multifocal glucagonomas

Journal name: Journal of Endocrinological Investigation

Author names: Carlo Smirne, Greta Maria Giacomini, Alessandro Maria Berton, Barbara Pasini, Francesca Mercalli, Flavia Prodam, Marina Caputo, Lodewijk Adriaan Anton Brosens, Edoardo Luigi Maria Mollero, Rosa Pitino, Mario Pirisi, Gianluca Aimaretti, Ezio Ghigo

Affiliation and e-mail address of the corresponding author: Department of Translational Medicine, University of Piemonte Orientale, 28100 Novara, Italy. Email: carlo.smirne@med.uniupo.it

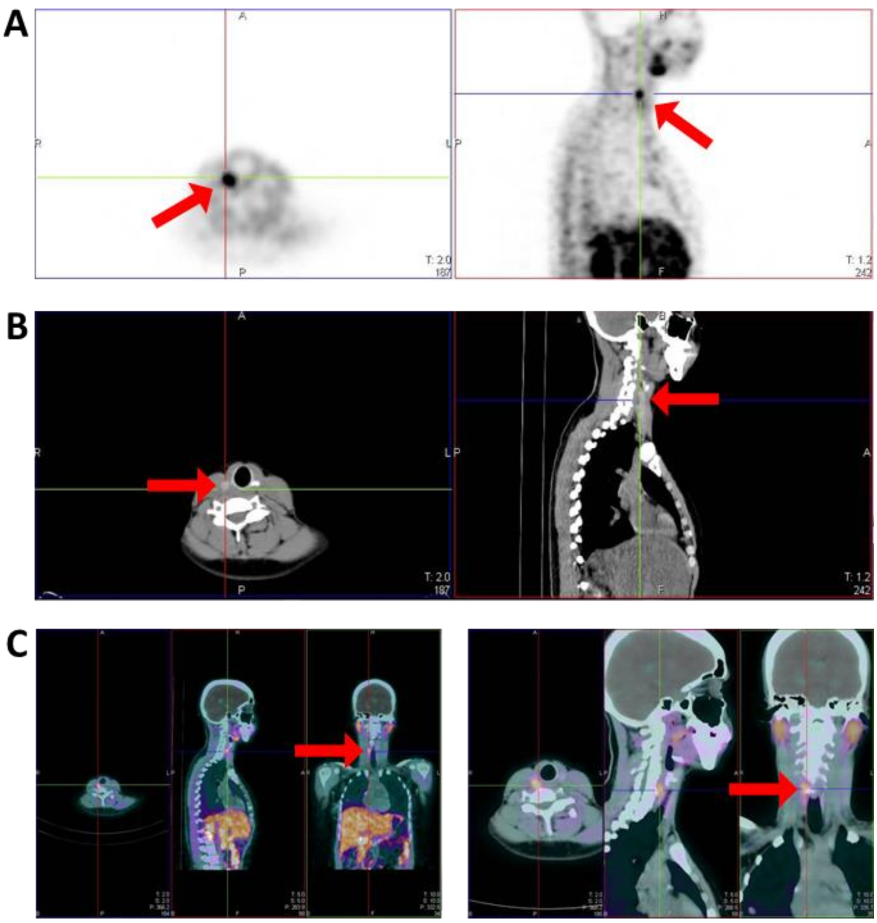

Supplement: Supplementary file 9 — Supplementary file9 (PDF 375 KB) [file 40618_2023_2287_MOESM9_ESM.pdf]
