# Supplementary material for: A novel likely pathogenetic variant p.(Cys235Arg) of the MEN1 gene in multiple endocrine neoplasia type 1 with multifocal glucagonomas
Source: J Endocrinol Invest. 2024 Jan 31;47(7):1815–25. doi: 10.1007/s40618-023-02287-x (PMC11196359; doi:10.1007/s40618-023-02287-x)
Supplement: Supplementary file 10 — Supplementary file10 (PDF 88 KB) [file 40618_2023_2287_MOESM10_ESM.pdf]

Online Resource 11 Main clinical findings of the index patient's daughter. Bold values are outside local laboratory normal ranges. **a** follow-up start **b** last available follow-up

Article title: A novel likely pathogenetic variant p.(Cys235Arg) of the *MEN1* gene in multiple endocrine neoplasia type 1 with multifocal glucagonomas

Journal name: Journal of Endocrinological Investigation

Author names: Carlo Smirne, Greta Maria Giacomini, Alessandro Maria Berton, Barbara Pasini, Francesca Mercalli, Flavia Prodam, Marina Caputo, Lodewijk Adriaan Anton Brosens, Edoardo Luigi Maria Mollero, Rosa Pitino, Mario Pirisi, Gianluca Aimaretti, Ezio Ghigo

Affiliation and e-mail address of the corresponding author: Department of Translational Medicine, University of Piemonte Orientale, 28100 Novara, Italy. Email: carlo.smirne@med.uniupo.it

The girl was born in Pakistan to unrelated parents with no issues. She underwent molecular analysis for the p.(Cys235Arg) variant of the *MEN1* gene at the age of 11 years, and since then she has been followed in a pediatric endocrinology outpatient clinic.
Currently (as of the date of publication of this manuscript) she is 13 years old, and she has always been in good health. She reports her periods started from the age of 12 years and are regular. In terms of symptoms she only complains of feeling tired and occasionally feels dizzy, but her mother checked her blood glucose on these occasions and she was not hypoglicemic. She reports sleep is fine and she has no constipation, abdominal pain, polyuria or polydipsia. On examination her thyroid is unremarkable. Her vitamin D was low and she has started oral supplementation since last visit. She will be seen again in one year's time after performing new blood tests and a first pituitary gland, adrenals and pancreas magnetic resonance imaging. Below are the results of the main laboratory tests at the beginning and at the end of the first two years of follow-up.

		**A. Follow-up start**	**B. Last follow-up**	**Local laboratory NR**
Blood tests	WBC	7.39	/	4.50-11.00 x10^9/L
	RBC	4.66	/	3.80-5.20 x10^{12}/L
	Hb	13.4	/	115-157 g/L
	PLT	284	/	150-450 x10^9/L
Liver function	ALT	16	/	0-40 U/L
	ALP	/	165	65-240 U/L
	Albumin	/	47	43-54 g/L
Renal function and Na/K	Cr	**47**	/	49.0-90.0 µmol/L
	eGFR	150	/	>90 mL/min/1.73m^2
	Na	142	/	133-146 mmol/L
	K	3.8	/	3.5-5.3 mmol/L
Glucose metabolism	FPG	4.44	/	3.9-5.6 mmol/L
	Insulin	13.8	/	6.0-27.0 µIU/mL
	C-peptide	2.16	/	0.80-4.20 ng/mL
Bone metabolism	Ca, total	2.37	2.42	2.15-2.50 mmol/L
	P	1.55	0.98	0.87-1.45 mmol/L
	Magnesium	/	0.78	0.70-1,00 mmol/L
	PTH	32.9	**45.7**	6.5-39.0 pg/mL
	25-hydroxy vitamin D	**4.3**	**23.7**	30.0-100.0 ng/mL
	Calcitonin	<1.0	/	1.0-14.0 pg/mL
Other hormones	Chromogranin A	<19.0	/	0.0-108.0 ng/mL
	GH	0.21	0.60	<10 ng/mL
	IGF-1	**626.3**	/	188.4-510.0 ng/mL
	PRL	206.3	/	59.4-619.0 mIU/L
	8 a.m. cortisol	**41**	/	45-240 µg/L
	ACTH	22.5	/	3.6-60.5 pg/mL
	TSH	0.728	**0.320**	0.450-3.500 mIU/L
	Free T4	15.3	18.6	11.4-22.7 pmol/L
	Free T3	6.4	5.4	3.5-6.4 pmol/L

ACTH, adrenocorticotrophic hormone; ALP, total alkaline phosphatase; ALT, alanine transaminase; Ca, calcium; Cr, creatinine; eGFR, estimated glomerular filtration rate; FPG, fasting plasma glucose; GH, growth hormone; Hb, hemoglobin; IGF-1, insulin-like growth factor 1; K, potassium; Na, sodium; NR, normal range; P, phosphorus; PLT, platelets; PRL, prolactin; PTH, parathyroid hormone; RBC, red blood cells; TSH, thyroid stimulating hormone; T4, thyroxine; T3, triiodothyronine; WBC, white blood cell; /, not tested.
