# Supplementary material for: A novel likely pathogenetic variant p.(Cys235Arg) of the MEN1 gene in multiple endocrine neoplasia type 1 with multifocal glucagonomas
Source: J Endocrinol Invest. 2024 Jan 31;47(7):1815–25. doi: 10.1007/s40618-023-02287-x (PMC11196359; doi:10.1007/s40618-023-02287-x)
Supplement: Supplementary file 12 — Supplementary file12 (PDF 92 KB) [file 40618_2023_2287_MOESM12_ESM.pdf]

Online Resource 1 Custom library used for next generation sequencing (NGS)[1] in our Laboratory, obtained with SureSelect-Custom Hereditary Cancer Solution probes (Agilent, USA). The total coverage was of 77 cancer genes, including the panel of 24 endocrine tumor genes tested in the current research (which are reported in bold type).

Article title: A novel likely pathogenetic variant p.(Cys235Arg) of the *MEN1* gene in multiple endocrine neoplasia type 1 with multifocal glucagonomas

Journal name: Journal of Endocrinological Investigation

Author names: Carlo Smirne, Greta Maria Giacomini, Alessandro Maria Berton, Barbara Pasini, Francesca Mercalli, Flavia Prodam, Marina Caputo, Lodewijk Adriaan Anton Brosens, Edoardo Luigi Maria Mollero, Rosa Pitino, Mario Pirisi, Gianluca Aimaretti, Ezio Ghigo

Affiliation and e-mail address of the corresponding author: Department of Translational Medicine, University of Piemonte Orientale, 28100 Novara, Italy. Email: carlo.smirne@med.uniupo.it

Gene symbol	Gene name
ACD	ACD shelterin complex subunit and telomerase recruitment factor
AIP	**aryl hydrocarbon receptor interacting protein**
APC	APC regulator of WNT signaling pathway
ATM	ATM serine/threonine kinase
AXIN2	axin 2
BAP1	**BRCA1 associated protein**
BARD1	BRCA1 associated RING domain 1
BMPR1A	bone morphogenetic protein receptor type 1A
BRCA1	BRCA1 DNA repair associated
BRCA2	BRCA2 DNA repair associated
BRIP1	BRCA1 interacting helicase 1
CDC73	**cell division cycle 73**
CDH1	cadherin 1
CDK4 (exon 2)	cyclin dependent kinase 4
CDKN1A	**cyclin dependent kinase inhibitor 1A**
CDKN1B	**cyclin dependent kinase inhibitor 1B**
CDKN2A	cyclin dependent kinase inhibitor 2A
CDKN2B	**cyclin dependent kinase inhibitor 2B**
CDKN2C	**cyclin dependent kinase inhibitor 2C**
CHEK2[2]	checkpoint kinase 2
CTR9	CTR9 homolog, Paf1/RNA polymerase II complex component
DICER1	dicer 1, ribonuclease III
DLST	**dihydrolipoamide S-succinyltransferase**
***DNMT3A* (exon 8)**	**DNA methyltransferase 3 alpha**
EPCAM	epithelial cell adhesion molecule
FH	**fumarate hydratase**
FLCN	**folliculin**
LZTR1	leucine zipper like transcription regulator 1
MAX	**MYC associated factor X**
MC1R	melanocortin 1 receptor
MDH2	**malate dehydrogenase 2**
MEN1	**menin 1**
MET (exons 16 to 19)	MET proto-oncogene, receptor tyrosine kinase
MITF (exon 9)	melanocyte inducing transcription factor
MLH1	mutL homolog 1
MSH2	mutS homolog 2
MSH3	mutS homolog 3
MSH6	mutS homolog 6
MUTYH	mutY DNA glycosylase
NF1	neurofibromin 1
NF2	NF2, moesin-ezrin-radixin like (MERLIN) tumor suppressor
NTHL1	nth like DNA glycosylase 1
PALB2	partner and localizer of BRCA2

PMS2[2]	PMS1 homolog 2, mismatch repair system component
POLD1 (exons 8 to 13)	DNA polymerase delta 1, catalytic subunit
POLE (exons 9 to 15)	DNA polymerase epsilon, catalytic subunit
POLE2 (exons 10 to 17)[2]	DNA polymerase epsilon 2, accessory subunit
POT1	protection of telomeres 1
PRKAR1A	**protein kinase cAMP-dependent type I regulatory subunit alpha**
PTCH1	patched 1
PTCH2	patched 2
PTEN[2]	phosphatase and tensin homolog
PTPN11	protein tyrosine phosphatase non-receptor type 11
RAD51C	RAD51 paralog C
RAD51D	RAD51 paralog D
REST	RE1 silencing transcription factor
RET	**ret proto-oncogene**
RPS20	ribosomal protein S20
SDHA[2]	**succinate dehydrogenase complex flavoprotein subunit A**
SDHAF2	**succinate dehydrogenase complex assembly factor 2**
SDHB	**succinate dehydrogenase complex iron sulfur subunit B**
SDHC	**succinate dehydrogenase complex subunit C**
SDHD	**succinate dehydrogenase complex subunit D**
SLC25A11	**solute carrier family 25 member 11**
SMAD4	SMAD family member 4
SMARCA4	SWI/SNF related, matrix associated, actin dependent regulator of chromatin, subfamily a, member 4
SMARCB1	SWI/SNF related, matrix associated, actin dependent regulator of chromatin, subfamily b, member 1
SPRED1	sprouty related EVH1 domain containing 1
STK11	serine/threonine kinase 11
SUFU	SUFU negative regulator of hedgehog signaling
TERF2IP	TERF2 interacting protein
TERT (promoter)	telomerase reverse transcriptase
TMEM127	**transmembrane protein 127**
TP53	tumor protein p53
TRIM28	tripartite motif containing 28
VHL	**von Hippel-Lindau tumor suppressor**
WT1	WT1 transcription factor

[1]NGS quality parameters: average coverage 1,234x; percentage of bases with at least 20 reads: 100%, percentage of bases with at least 50 reads: 100%; number of reads in regions covered by DNA sequencing: 2,903,124 (mapped reads). Sequencing made with the MiSeq platform (Illumina, San Diego, CA, USA); sequence alignment, variant calling and Copy Number Variants (CNVs) analysis performed with DDM software (Sophia Genetics, Saint Sulpice, Switzerland) (reference genome: GRch37/hg19 from Genome Reference Consortium), filtering for variant quality, allelic frequency (GnomAD < 0.5% for dominant) and consequence of the substitution.

[2]The following coding regions were excluded from the deletion/duplication analyses: *CHEK2* exon 7, *POLE2* exon 13, *PTEN* exon 3, *PMS2* exons 12 to 15, *SDHA* exons 13 to 15
